# Vanishing clear cell carcinoma of the kidney presenting with skin metastases – a case report

**DOI:** 10.3332/ecancer.2025.1959

**Published:** 2025-08-07

**Authors:** Sidhart Misra, Zainab Yusufali Motiwala, Ayyaz Mulla, Jagatheswaran Chinnathambi, Danny Darlington Carbin

**Affiliations:** 1 Armed Forces Medical College, Pune 411040, India; 2 Medical Intern, JNMCH, Aligarh Muslim University, Aligarh 202001, India; 3 United Hospital, Jayanagar, Bengaluru 560011, India; 4 Assistant Professor of Urology, Manakkula Vinayagar Medical College, Pondicherry 605107, India; 5 Locum Consultant Urologist, Ashford and St Peters NHS Foundation Trust, Chertsey, UK

**Keywords:** renal cell carcinoma, metastasis, unknown primary neoplasms, lung, liver, skin

## Abstract

Renal cell carcinoma (RCC) is one of the common genitourinary malignancies that has an increasing incidence. RCC presents a diagnostic challenge due to its wide range of clinical manifestations, often leading to delays in diagnosis and complicating management strategies. These tumours have clear cells in 70% of cases and have a high preponderance of haematogenous metastases to distant organs such as lungs, bones and liver. Skin metastases of RCC in the absence of an obvious renal tumour are rare. We report a young woman with clear cell renal carcinoma with skin metastasis who presented with left loin pain and acute kidney injury, prompting a series of comprehensive investigations, including imaging studies and laboratory tests. Despite these efforts, a primary tumour remained elusive. However, a breakthrough occurred when histopathological examination of a skin nodule biopsy, alongside cytological analysis of nephrostomy fluid, ultimately identified the underlying cause as malignant RCC. Despite commencing palliative Sunitinib therapy based on intermediate risk criteria, the patient died from lung metastases after 6 months of systemic medication. Here is a more succinct version. This case report emphasises the need to investigate renal primaries in unknown-origin metastases and the importance of a thorough diagnostic approach for RCC.

## Introduction

Renal cell carcinoma (RCC) predominates as the primary type of urogenital cancer, showing a greater prevalence in men than in women, and it carries a mortality rate ranging from 30% to 40% [1]. For RCC, metastasis rates were noted as 3.6% for tumours measuring ≤4 cm, 13.1% for tumours ranging from 4 to 7 cm, 30.3% for those between 7 and 10 cm and 45.1% for tumours larger than 10 cm [2]. Bone metastasis is common, followed by brain, pancreas, adrenal gland, gallbladder, liver and lymph nodes [3, 4]. RCC presenting with skin metastases is uncommon but not a rare occurrence. The incidence of skin metastases in patients with RCC ranges from 3.3% to 6.8% [5, 6]. The timing of metastases could be before, after or at the same time as the diagnosis of the primary renal cell carcinoma [5, 7]. It has been observed that the overall prognosis of patients with RCC and skin metastases is generally poor with survival ranging from 7 to 36 months after detection [5–7].

Vanishing tumours are lesions that disappear or reduce in size spontaneously without treatment. Some of the common locations include the brain and kidneys. In the brain, two common diagnoses for vanishing tumours are malignant tumours or multiple sclerosis [8]. In the kidney, the main associations with renal cell carcinoma are malignant peripheral nerve sheath tumours and xanthogranulomatous pyelonephritis [9, 10]. There have been reports of vanishing tumours in cases of cerebral metastases of unknown primary [11].

This case study presents a unique case of cutaneous metastases in a patient with vanishing RCC. The interesting rare aspect of this case was that the renal tumour underwent complete regression due to spontaneous necrosis and disappeared. As a consequence, the tumour was not detected on imaging. However, the biopsy of the skin lesion revealed clear cell RCC, which is rather unusual. The regression of the renal mass combined with the confirmation of RCC on skin biopsy makes this case a rare occurrence.

## Case presentation

This is a case of a 45-year-old woman who presented to our emergency department with left loin pain and acute kidney injury (AKI). She had a past medical history of diabetes but otherwise, she was generally fit. On examination, she was febrile with stable vital signs and her performance status was 0. Physical examination was positive for tenderness in the left loin region and a soft abdomen on palpation. Her hematological investigations revealed low hemoglobin, elevated white cell count and thrombocytopenia. Other relevant findings include hypoalbuminemia and hypocalcemia. Collectively, the patient was diagnosed with stage 3 AKI.

**Figure 1.**
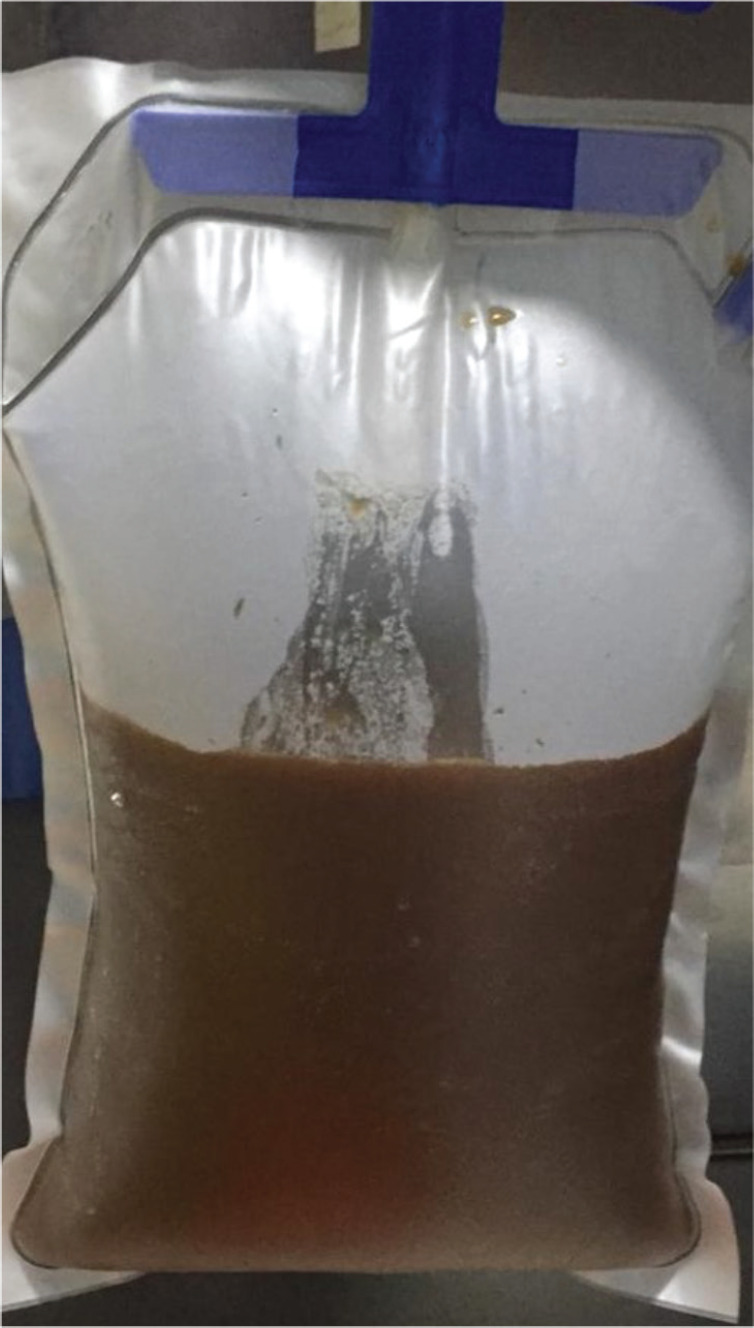
Left-sided nephrostomy drained brown hemo-serous fluid.

**Figure 2.**
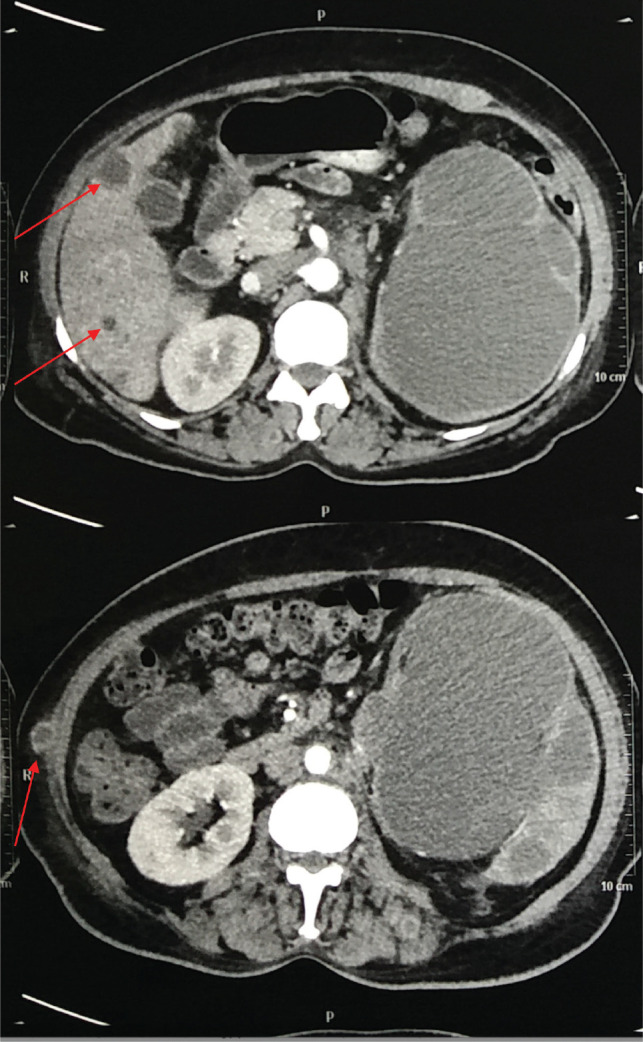
Contrast-enhanced CT scan of the abdomen and pelvis (Transverse section) representing liver and skin metastasis (red arrow).

**Figure 3.**
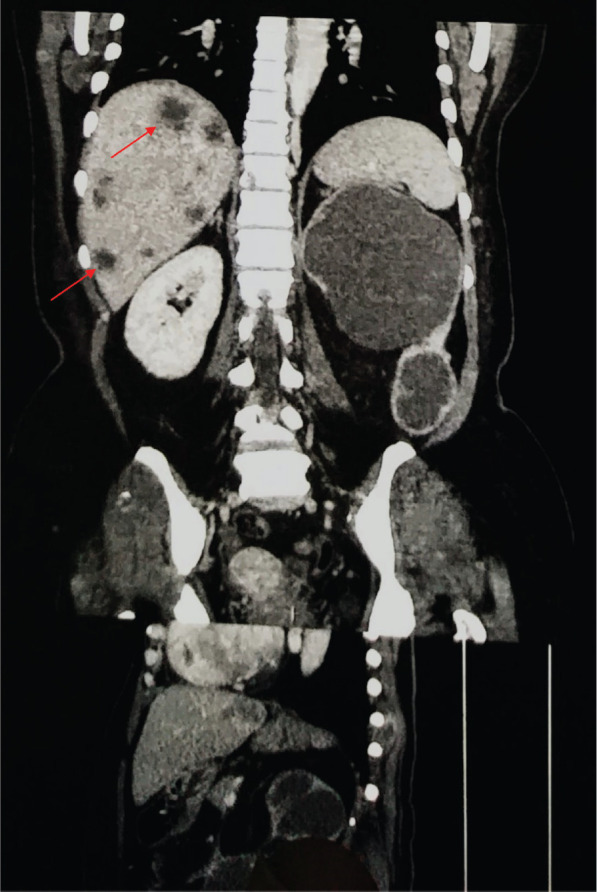
Contrast-enhanced CT scan of the abdomen and pelvis (Coronal section) representing metastasis (red arrows).

**Figure 4.**
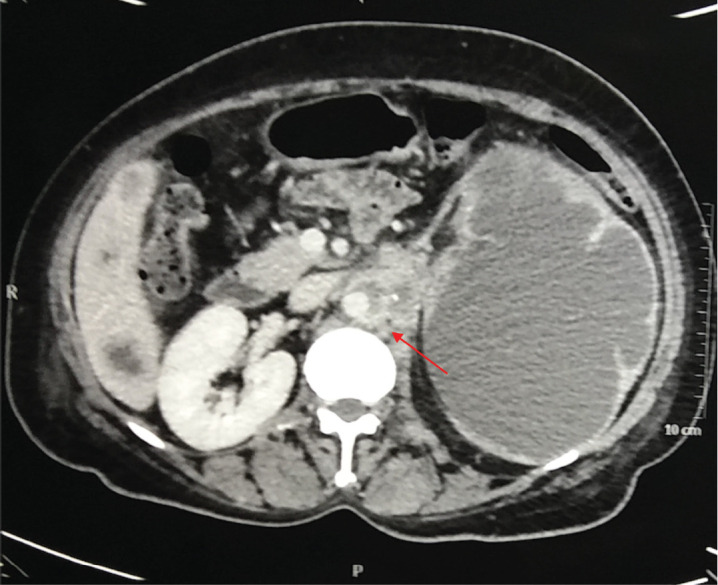
CT scan of the abdomen and pelvis (Transverse section) representing para aortic lymphadenopathy (red arrow).

An urgent non-contrast CT scan showed a left hydronephrotic kidney, with no renal parenchyma. With the patient being in sepsis, a prompt left-sided nephrostomy was done which drained brown fluid (Figure 1). The cytological analysis of the fluid revealed pleomorphic malignant clear cells with abundant nucleoli and nuclear atypia. Following the resolution of the acute kidney injury, a contrast-enhanced CT scan was done which showed multiple liver and lung metastasis (Figures 2 and 3), as well as para-aortic lymphadenopathy (Figure 4). In addition, several skin nodules were noted. On examination, one of the nodules was located in the right hypochondrium which was mobile, hard and measured 3 × 4 cm. Subsequently, a biopsy was taken under local anesthesia and the histology confirmed clear cell RCC. Considering the lesion to be intermediate risk based on the parameters given by the International Metastatic Renal Cell Carcinoma Database Consortium (IMDC) such as platelet count, albumin, calcium, prior nephrectomy, haemoglobin and metastatic presentation, the patient was deemed unsuitable for nephrectomy [12, 13]. The multidisciplinary oncology team decided to initiate palliative Sunitinib therapy [14–16]. Despite receiving palliative treatment for 6 months, the patient succumbed to complications arising from lung metastasis and passed away due to acute massive hemoptysis.

## Discussion

RCC is a heterogeneous group of cancers arising from renal tubular epithelial cells, with clear cell renal cell carcinoma (ccRCC) originating from proximal tubules with a histological presentation of thin-walled cells filled with lipids and glycogen [17, 18]. It is a common urinary tract malignancy that has witnessed a rise in incidence worldwide [19]. At a global level, the incidence and mortality have been reported to be 434,840 and 155,953, respectively, according to WHO [20]. Higher rates have been noticed in developed countries [21]. 15% of clear cell carcinomas have the potential to metastasise [22, 23]. The majority of ccRCC cases originate from mutations in the Von Hippel-Lindau gene, which is located on the short arm of chromosome 3 [24].

Carcinoma of unknown primary accounts for about 15% of all solid tumours, and it poses a diagnostic challenge [25]. To determine its primary site, immunohistochemistry (IHC) staining patterns, particularly cytokeratin 7 and 20 play a crucial role [26]. They use specific antibody panels to identify the various skin cancers such as melanoma, lymphoma and sarcomas. In addition, they aim to determine the primary sites of the tumour (breast, prostate and thyroid). CK7+/CK20+ marker is commonly observed in urothelial cancers [25]. In instances where the primary origin remains unknown even after IHC analysis, additional diagnostic procedures such as cytopathological specimens and gene expression studies can be carried out [26, 27].

Lung involvement of metastatic ccRCC accounts for approximately 20%–30% of metastatic occurrences with a projected 5-year survival rate ranging from 21% to 60% in pulmonary metastasectomy cases, in contrast to 11% for non-operated patients [28]. Lung metastasis has also been reported in a patient 16 years post radical nephrectomy [29]. This emphasis is on the high post nephrectomy recurrence rate within the lungs which are the most common site for distant metastasis (50%–60%).

This case report describes a very rare instance of metastatic RCC (mRCC) presenting without an obvious primary renal mass/tumour and the histology of the skin lesions confirmed the origin of the tumour. This histological diagnosis is related to an important phenomenon called Azzopardi effect which was first described in 1959 [30]. It is defined as the deposition of basophilic nuclear material in the blood vessel wall which shows a positive feulgen reaction suggestive of DNA material. It has been described in various types of cancer such as Burkitt lymphoma, small cell carcinoma, Merkel cell carcinoma and medulloblastoma [31]. The underlying mechanism behind this phenomenon is the release of nucleic acid from degenerating cancer cells. It has been noticed more commonly with testicular; cancer however, our case depicted the Azzopardi effect with mRCC which is a rare occurrence. Additionally, spontaneous primary tumour regression is also a rare phenomenon observed in this case that can be attributed to the different mechanisms like tumour necrosis, angiogenesis inhibition and apoptosis [32].

The given case demonstrated metastasis to the skin, liver and, lung with no primary tumour identified. A similar case was reported by Razi et al [33] demonstrating the presence of mRCC in para-aortic lymph node where the origin of the tumour could not be determined. The IHC of the node was positive for CK20, CD10, AMACR, PAX8, CAIX and CK8/18 which indicated renal origin. Another case report demonstrated biopsy proven RCC in the bones like scapula, ribs and pelvis with no evidence of the primary tumour [34]. Furthermore, a study presented 2 cases of mRCC to the pubo-iliac bone and knee, with no parenchymal solid renal lesion observed on CT imaging [35]. The diagnostic challenges presented by mRCC of unknown primary origin and the role of IHC in diagnosis were highlighted in the cases.

Skin metastases originating from RCC manifested as nodular growths that proliferate swiftly, exhibiting round or oval shapes with varying colours from skin tone to hues of red-purple [36]. The IMDC criteria are considered the standard for stratifying risk in mRCC patients [37]. For individuals classified as intermediate- or poor-risk, the preferred first-line therapies include ipilimumab + nivolumab or pembrolizumab + axitinib, while alternative options such as avelumab/axitinib and targeted therapy (sunitinib or pazopanib) are available, particularly for patients unsuitable for or intolerant to combination therapy or immunotherapy [12]. Interleukin-2 or interferon alfa are commonly employed as primary therapies for mRCC due to its high resistance to chemotherapy [38]. Sunitinib, a tyrosine kinase inhibitor with multi-targeted activity against VEGFR, PDGFR, c-Kit, RET and Flt3, demonstrates notable efficacy as a first-line treatment option for patients diagnosed with mRCC as evidenced by higher response rates, prolonged progression-free survival and improved overall survival [39, 40]. Other treatment options for mRCC include surgical debulking and radiotherapy [41].

The prognosis depends on the extent of metastatic spread and it is associated with poor outcomes in cases of spread to the lung, liver and skin as seen in the given case. Risk stratification with IMDC criteria and using IHC for histological confirmation are essential for initiation of appropriate therapy [42]. Therefore, this case highlights the need for further research on this rare phenomenon and the need for standardisation of management strategies. Additionally, in the era of immunotherapy, it is ideal to get a biopsy with immunohistochemistry to identify the protein expression as well as molecular and cytogenetics studies that can guide future personalised treatments [20].

## Conclusion

Clinicians should have a high degree of suspicion for RCC, especially in patients with carcinoma of unknown origin. Skin metastases should be considered as a potential manifestation of RCC even in the absence of an obvious renal mass. When considering RCC in the context of unexplained metastatic disease, cytological analysis of fluids such as nephrostomy fluid can also provide important insights.

## Conflicts of interest

None.

## Funding

None.

## Informed consent

Informed consent was obtained for anonymous publication of the images, surgical videos, details of the clinical presentation, surgery and follow-up.

## Author contributions

SM- manuscript writing, data collection, literature search, ZYM- manuscript writing, data collection, literature search, AM- manuscript writing, data collection, literature search, images, primary surgical team, JC- manuscript review, literature search, primary surgical team, follow up, images, DDC- manuscript review, data collection, literature search, primary surgeon.
